# Capture efficiency and trophic adaptations of a specialist and generalist predator: A comparison

**DOI:** 10.1002/ece3.2812

**Published:** 2017-03-21

**Authors:** Ondřej Michálek, Lenka Petráková, Stano Pekár

**Affiliations:** ^1^ Department of Botany and ZoologyFaculty of ScienceMasaryk UniversityBrnoCzech Republic

**Keywords:** Araneae, Araneophagy, cannibalism, *Drassodes*, *Lampona*, morphology, NGS, trophic niche

## Abstract

Specialist true predators are expected to exhibit higher capture efficiencies for the capture of larger and dangerous prey than generalist predators due to their possession of specialized morphological and behavioral adaptations. We used an araneophagous spider (*Lampona murina*) and a generalist spider (*Drassodes lapidosus*) as phylogenetically related model species and investigated their realized and fundamental trophic niches and their efficacy with respect to prey capture and prey handling. The trophic niche of both species confirmed that *Lampona* had a narrow trophic niche with a predominance of spider prey (including conspecifics), while the niche of *Drassodes* was wide, without any preference. DNA analysis of the gut contents of *Lampona* spiders collected in the field revealed that spiders form a significant part of its natural diet. *Lampona* captured significantly larger prey than itself and the prey captured by *Drassodes*. As concerns hunting strategy, *Lampona* grasped the prey with two pairs of legs possessing scopulae, whereas *Drassodes* immobilized prey with silk. *Lampona* possess forelegs equipped with scopulae and a thicker cuticle similar to other nonrelated araneophagous spiders. *Lampona* fed for a longer time and extracted more nutrients than *Drassodes*. We show that specialized behavioral and morphological adaptations altogether increase the hunting efficiency of specialists when compared to generalists.

## 1 Introduction

Optimal foraging theory assumes that the choice of the food depends on the net energetic gain. Generalists spend less energy on foraging, as they readily accept most of the food they encounter. Their handling time is relatively short. Specialists spend more time and energy on searching for food, the handling time is relatively long, and the energy gain from a single food item is relatively big (Davies, Krebs, & West, 2012; Townsend, Begon, & Harper, 2003).

Trophic adaptations have been studied mostly in herbivores, parasites, and parasitoids (Futuyma & Moreno, 1988). A dichotomy in foraging between generalists and specialists has been shown mainly in herbivores (Bernays, Singer, & Rodrigues, 2004). True predators (i.e., predators that hunt more than one prey item during their lifetimes) have not been studied intensively in this regard. It appears that true predators are less frequently specialized than herbivores, parasites, and parasitoids (Thompson, 1994), as the great majority of them are generalists, catching prey smaller than themselves (Griffiths, 1980). Specialist predators seem to specialize on relatively large prey (e.g., Bulbert, Herberstein, & Cassis, 2014; Mori & Vincent, 2008; Pekár, Šedo, Líznarová, Korenko, & Zdráhal, 2014; Yamada & Boulding, 1998). Such prey is overcome by the use of specialized adaptations, especially by the ability to perform effective prey capture behaviors or by the possession of the right ‘tools’ to capture prey (Ferry‐Graham, Bolnick, & Wainwright, 2002).

Such behavioral adaptations include a variety of hunting tactics which are effective in hunting successfully for different prey types (e.g., Harland & Jackson, 2006). Specialists usually possess hunting strategies dedicated to subduing their focal prey. For example, web‐invading araneophages deceive their victims by imitating ensnared prey (e.g., Jackson, 1990) or the male's courtship signals (Jackson & Wilcox, 1990). Myrmecophagous spiders preying on ants use one of three different approaches to capture ants depending on their size and dangerousness (Pekár & Toft, 2015).

Morphological traits may also affect handling effectiveness. For example, specialized crabs feeding on molluscs possess broad claws with dull protuberances, which are well adapted to exerting greater pressure and to breaking hardened shells (Yamada & Boulding, 1998). Snakes specialized on frogs are able to open their jaws wider for the easier swallowing of such bulky prey (Mori & Vincent, 2008). Crustaceophagous spiders of the genus *Dysdera* have evolved variously shaped fangs to penetrate the defences of woodlice (Řezáč, Pekár, & Lubin, 2008). Araneophagous spiders of the genus *Palpimanus* use stout legs with scopulae to firmly grasp their prey. They also have a thick cuticle preventing counter attack by the dangerous prey they catch (Pekár, Šobotník, & Lubin, 2011).

**Figure 1:**
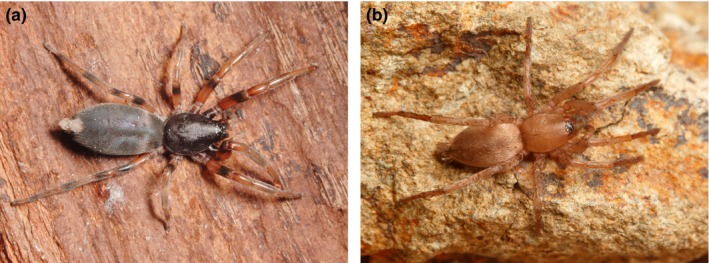
(a) *Lampona murina*, female. (b) *Drassodes lapidosus*, juvenile. (Photograph: O. Michálek)

The aim of this study was to compare the capture efficiencies and associated adaptations of a specialist and a generalist predator. We chose spiders as our model system, as both generalist and specialist species can be found among these true predators (Pekár, Coddington, & Blackledge, 2012). First, we investigated the trophic niche of two phylogenetically related species, *Lampona murina* L. Koch, 1873 (Lamponidae) (Figure 1a), and *Drassodes lapidosus* (Walckenaer, 1802) (Gnaphosidae) (Platnick, 2000) (Figure 1b), to support our expectation that they represent a specialist and a generalist, respectively. *Lampona* is an Australian genus reported to be araneophagous, as it was often observed invading the webs of other spiders (Platnick, 2000). *Drassodes* is a Palearctic genus also reported to occasionally catch spiders (Chinery, Morris, & Hughes, 1979). We expected *Lampona* to have higher capture and handling efficiencies than *Drassodes* due to its possession of specialized behavioral and morphological hunting adaptations.

## 2 Materials and Methods

### 2.1 Spiders

*Lampona murina*, further shortened to *Lampona*, (*N* = 104) were collected under the bark of *Eucalyptus* trees on the Macquarie University campus, North Ryde, Sydney, Australia, between February and July 2014. Of these, 47 individuals were placed immediately into pure ethanol to be used in gut content analysis. A total of 57 individuals (prosoma length 2.78 ± 0.86 mm) were kept alive for laboratory experiments.

*Drassodes lapidosus*, further shortened to *Drassodes*, (*N* = 87, prosoma length 2.87 ± 0.77 mm) were collected under stones at a former limestone quarry in Hády, Brno, Czech Republic, between March and July 2014.

All spiders used in laboratory experiments were kept in plastic vials containing moisturized gypsum, which were placed in a chamber at a constant temperature (27 ± 1°C) and under a LD regime (16:8). Spiders were fed at least once a week with a cricket or a spider or were allowed to consume the prey accepted in trials. Experiments were performed from April to December 2014.

All analyses were performed using R environment (R Core Team, 2014).

### 2.2 Fundamental niche

To investigate the fundamental trophic niche, acceptance trials were performed. A total of 55 individuals of *Lampona* and 35 individuals of *Drassodes* were used. Spiders were starved for 1 week before using them in trials. Individuals were placed singly in Petri dishes (diameter 5 cm). The trials began after at least 1 hr of acclimation. Each of 13 prey types (Table 1) was offered to a spider in a randomized order. If the prey was not attacked within 10 min of coming into contact with the predator, it was replaced with a different prey type. The trial ended when a spider killed and consumed a prey. If a spider did not accept any prey type, it was considered unmotivated to eat (i.e., satiated or preparing to molt), and data concerning such individuals were rejected. Trials were performed approximately in one‐week intervals for each individual. Each prey type was offered to individual spiders only once.

**Table 1:** List of prey types used in acceptance experiments, their body size (*body size, **prosoma size), and the number of offered prey

Order/family	Species	Prey size (mm)		***N***	
				*Drassodes*	*Lampona*
Isopoda	*Porcellio scaber* (Latreille, 1804)	5.68 ± 1.83	*	26	19
Araneae: Lycosidae	*Pardosa* sp.	3.05 ± 1.55	**	29	39
Araneae: Thomisidae	*Misumena vatia* (Clerck, 1757)	1.25 ± 0.47	**	22	33
	*Xysticus* sp.				
Collembola	*Orchesella* sp.	2.78 ± 0.74	*	22	20
Dictyoptera: Blattellidae	*Symploce pallens* (Stephens, 1835)	5.12 ± 1.75	*	28	29
Isoptera	*Reticulitermes santonensis* (Feytaud, 1924)	3.70 ± 1.77	*	20	27
Ensifera	*Acheta domestica* (Linnaeus 1758)	5.09 ± 2.28	*	22	‐
	*Gryllus assimilis* (Fabricius, 1775)		*	‐	34
Auchenorrhyncha	gen. et sp. indet.	4.16 ± 1.01	*	24	23
Heteroptera	*Polymerus unifasciatus* (Fabricius, 1794)	4.71 ± 0.43	*	22	‐
	*Daerlac nigricans* (Distant, 1918)	7.36 ± 0.59	*	‐	32
Lepidoptera: Geometridae	Caterpillars, gen. et sp. indet.	11.76 ± 4.08	*	23	15
Hymenoptera: Formicidae	*Lasius niger* (Linnaeus, 1758)	3.52 ± 0.32	*	28	‐
	*Lasius flavus* (Fabricius, 1781)		*		
	*Polyrhachis vermiculosa* (Mayr, 1876)	6.67 ± 0..49	*	‐	15
Diptera	*Drosophila melanogaster* (Meigen, 1830)	2.05 ± 0.22	*	23	21
	*Drosophila hydei* (Sturtevant, 1921)		*		
Coleoptera	*Tribolium castaneum* (Herbst, 1797)	3.80 ± 0.75	*	20	26

The acceptance (i.e., the relative frequencies of acceptance) of each prey type was compared to the average prey acceptance of *Drassodes* and *Lampona* using a linear model with arcsine square root transformation of the data and sum contrasts. The standardized Levins’ index (*B*_*A*_) of niche breadth (Hurlbert, 1978) was used to calculate the fundamental trophic niche breadth of both *Lampona* and *Drassodes* using the following formula:

$$B_{A}=\frac{\left[\left(\frac{1}{\sum_{i=1}^{n}{p_{j}}^{2}}\right)-1\right]}{\left(n-1\right)}$$

where *p*_*j*_ is the proportion of individuals accepting the *j*th prey, and *n* is the total number of prey types. Values of *B*_*A*_ higher than 0.6 indicate a wide niche, and values below 0.4 indicate a narrow niche (Novakowski, Hahn, & Fugi, 2008). Twelve prey types instead of 13 were used to calculate the index, as all spiders (Lycosidae and Thomisidae) were pooled and considered as a single category. Pianka's index (*O*_*jk*_) (Pianka, 1973) was used to calculate niche overlap using the following formula:

$$O_{jk}=\frac{\sum_{i=1}^{n}p_{ij}p_{ik}}{\sqrt{\sum_{i=1}^{n}{p_{ij}}^{2}\sum_{i=1}^{n}{p_{ik}}^{2}}}$$

where *p*_*ij*_ is the proportion of the *i*th prey in the diet of *j*th species, *p*_*ik*_ is the proportion of the *i*th prey in the diet of *k*th species, and *n* is the total number of prey types. Values close to one indicate high overlapping; a zero value represents excluded niches.

### 2.3 Realized niche of *L. murina*

The realized trophic niche was investigated only in the specialist (*Lampona*), as examining the realized niche in the generalist (*Drassodes*). We assumed that the breadth of the realized niche of the generalist would be wide and only wanted to confirm the narrow niche in the specialist. The niche breadth was investigated using gut contents and analyzed using next‐generation sequencing (NGS). General invertebrate primers (Zeale, Butlin, Barker, Lees, & Jones, 2011) were used for the amplification of prey DNA from the guts of *Lampona* spiders. As these primers allow the amplification of a wide range of taxa, including spiders, and the predators’ DNA prevails over prey DNA in the predators’ opisthosoma, it was necessary to design blocking oligos (Vestheim & Jarman, 2008) which would prevent the amplification of *Lampona* spiders’ DNA but allow the amplification of their potential prey, including closely related spiders. DNA was extracted from the tibia of two *Lampona murina* individuals and from other spiders (*Clubiona robusta* L. Koch, 1873*, Clubiona* sp., *Euryopis* sp., *Euryopis umbilicata* [L. Koch, 1972], *Hemicloea* spp., *Holoplatys planissima* [L. Koch, 1879], *Ocrisiona* spp., *Sandalodes superbus* [Karsch, 1878]*, Servaea incana* [Karsch, 1878], *Myrmarachne luctuosa* [L. Koch, 1879], *Myrmarachne erythrocephala* [L. Koch, 1879]), ants, and true bugs using DNeasy Blood & Tissue Kit (Qiagen) following the manufacturer's protocol. All these taxa represented invertebrates that occur sympatrically with *L. murina*. The cytochrome c oxidase I gene fragment was PCR‐amplified using LCO (5′‐ GGTCAACAAATCATAAAGATATTGG‐3′) and HCO (5′‐TAAACTTCAGGGTGACCAAAAAATCA‐3′) primers (Folmer, Black, Hoeh, Lutz, & Vrijenkoek, 1994) in all samples. The reaction mixture consisted of 5 μl of DNA, 1 μl of each primer (10 μmol/L), 0.4 μl of 10 mmol/L dNTPs, 2.2 μl of 25 mmol/L MgCl_2_, 2 μl of 10× PCR buffer, 1 μl of bovine albumin serum (BSA), 0.3 μl of Taq polymerase (5u/μl), and 7.1 μl of DNA‐free water. The PCR conditions were as follows: initial denaturation at 94°C for 3.5 min; 38 cycles of 94°C for 1 min, 46°C for 1 min, 72°C for 1.5 min; and a final extension at 72°C for 7 min. PCR products were detected by electrophoresis in 2% GoodView‐stained agarose gels. Amplified products were purified using the QIAquick PCR Purification Kit (Quiagen) and sequenced in both directions with BigDye Terminator v3.1 Sequence Kit (Applied Biosystems). Sequencing was carried out on an ABI Prism 3130 Genetic Analyzer (Applied Biosystems). Sequences were assembled in Sequencher 4.8 (Gene Codes Corporation, Ann Arbor, MI) and aligned using ClustalW (Thompson, Higgins, & Gibson, 1994) implemented in MEGA 5.1 (Tamura et al., 2011). A reverse blocking primer was designed using Amplicon.b08 (Jarman, 2004): A unique section of 23 bases, typical for *Lampona* only, which was situated inside the fragment amplified using general invertebrate primers and closer to its 3′ end, was selected and modified with C3 spacer. The blocking primer (BlkLamp, 5′‐CGTCACCTAATAATCTACCGGAC‐3′) was tested with all potential prey samples and with *Lampona* spiders both to ensure that none of the potential prey would be blocked and to unify optimal PCR conditions.

In two adult males, ten adult females, and 35 juveniles of *L. murina*, prey DNA was extracted from their opisthosoma using the same approach as above, with a change in the final step when only 50 μl of elution buffer was added. PCR reactions with general invertebrate primers (ZBJArtF1c: 5′‐AGATATTGGAACWTTATATTTTATTTTTGG‐3′, ZBJArtR2c: 5′‐WACTAATCAATTWCCAAATCCTCC; Zeale et al., 2011) were performed using Multiplex PCR Kit (Qiagen) under the following conditions: initial denaturation at 95°C for 15 min; 42 cycles of 94°C for 30 s, annealing temperature 53.6°C for 90 s, 72°C for 90 s; and a final extension at 72°C for 10 min. The reaction mixture consisted of 10 μl of Multiplex PCR Master Mix, 1.5 μl of Q‐Solution, 2.5 μl of RNase‐free water, 0.5 μl of 10 μmol/L forward and 0.5 μl of reverse primers, 1 μl of 100 μmol/L blocking oligo, and 7 μl of DNA. Each sample was PCR‐amplified using a unique combination of primers tagged with MID identifiers (10 base‐long barcoding sequences; we used seven different MIDs on the forward primer and another seven MIDs on the reverse primer, which allowed us to assign all DNA reads to the predator individuals). PCR products were detected by electrophoresis in 2% GoodView‐stained agarose gels. When no PCR product was detected, PCR was repeated until DNA was available. PCR products were purified using QIAquick PCR Purification Kit (Qiagen) according to the manufacturer's protocols. The concentration of each PCR product was measured using a NanoDrop 8000 UV‐Vis Spectrophotometer (Thermo Scientific). 5 μl of each 50 μg/μl PCR product was pooled into the same sterile vial and sent for sequencing. Enrichment (emPCR) and sequencing on an Ion Proton System with an Ion 316 chip with 400‐base read‐length chemistry were provided by the SEQme company (Dobříš, Czech Republic).

The sequencing output was processed using the Galaxy platform (https://usegalaxy.org/), BioEdit 7.2.5 (Hall, 1999), MEGA 5.10 (Tamura et al., 2011), fastx‐toolkit, and the EMBOSS package (Rice, Longden, & Bleasby, 2000). Reads were split according to their MIDs, resulting in files corresponding to predator individuals. Then, forward and reverse primers were removed, and reads were filtered according to their length; reads shorter than 120 bases were removed. The reads were collapsed, and rare haplotypes (containing <2 identical reads) were removed. Sequences which contained stop codons were removed; the remaining were aligned using MAFFT (Katoh & Standley, 2013), and the sequences with indels causing frameshifts were also removed. The remaining haplotypes were clustered into MOTUs (=molecular operational taxonomic units) using jMOTU 4.1 (Jones, Ghoorah, & Blaxter, 2011) with a 4‐bp cutoff. Each MOTU was compared to the GenBank database (http://blast.ncbi.nlm.nih.gov/Blast.cgi) using megablast, to the BOLD database (http://www.boldsystems.org/), and to the sequences obtained for the primer design. When a sequence was more than 99% identical to that of a certain species, the prey was identified as that species. Similarly, a sequence which was more than 98% identical to those of several species belonging to a single genus was assigned to that genus. By the same token, similarity to several genera from one family allowed classification to a family level and several families to an order. Three different haplotypes of *Lampona murina* were distinguished in the gut, which allowed us to detect cannibalism. Therefore, we have additionally extracted DNA from tibia of each *Lampona* individual and sequenced the cytochrome c oxidase gene (as described above) to determine whether the sequences found in guts belonged to the predator or to its conspecific prey. If the haplotype found in predator's gut was identical to the haplotype obtained from tibia of the same predator individual, we concluded that only predator's DNA was obtained. If the haplotype found in predator's gut differed from the predator's haplotype (obtained from tibia), we concluded that the predator fed on conspecific prey.

Obtained valid sequences for prey were transferred to binary data, that is, the presence of a prey sequence type in *Lampona* individuals. Identified prey was assigned to order level. To correct for the effect of secondary predation (i.e., the detection of prey consumed by the prey of *Lampona*), prey amplified together with other predator (insect prey together with spider prey other than *Lampona*, spider prey together with *Lampona* prey, and *Lampona* prey together with other *Lampona* prey) in one individual was multiplied by the relative capture frequency for the particular order obtained from the acceptance experiments. Specifically, Lepidoptera and Coleoptera prey were multiplied by zero as they were rejected in acceptance experiments; Diptera prey was multiplied by 0.09; spider prey was multiplied by the mean relative frequency of two spider types used (Lycosidae and Thomisidae, 0.775); and unidentified arthropods and prey not used in the experiments (Psocoptera) were multiplied by the overall mean of the relative frequency (0.26), as these categories could not be assigned to a certain prey type used in the acceptance experiments. From the corrected data, the total number of spider prey was compared to the total number of other prey using the chi‐square test, and the standardized Levins’ index of niche breadth (*B*_*A*_) was used to calculate the realized trophic niche breadth.

### 2.4 Capture efficiency and capture behavior

To compare the hunting efficiencies of *Lampona* and *Drassodes* for differently sized prey, spiders (*Pardosa*,* Alopecosa*, Lycosidae, Araneae) of various sizes were offered to both species in a similar manner as in the acceptance trials. Individuals of *Lampona* and *Drassodes* were placed singly in Petri dishes, and after acclimation, the prey was offered. If the prey was not accepted within 10 min of the predator and prey coming into contact, it was replaced by a smaller one. The fangs of large *Alopecosa* spiders were glued by a droplet of cyanoacrylate superglue before the trial to prevent counter attack. The length of the prosoma of all spiders, both predators and prey, was measured under a LEICA EZ5 binocular lens with an ocular micrometer before experiments. In total, 143 trials with 46 individuals of *Lampona* and 76 trials with 31 individuals of *Drassodes* were performed. The logit model with binomial distribution and generalized estimating equations (GEE) from the geepack package (Halekoh, Højsgaard, & Yan, 2006) was used. GEE is an extension of GLM for correlated data. It was used because there were repeated measurements on each individual of *Lampona* and *Drassodes*. A binomial error structure and exchangeable correlation structure were used.

To compare the hunting strategies of both species, capture sequences were recorded using a high‐speed camera (IDT MotionXtra N3) at 500 fps. In several cases, a lower frame rate (200 fps) was used in order to record the whole hunting sequence. A high‐speed camera was used, as the hunting actions of both *Lampona* and *Drassodes* were very quick: Prey capture took only a few seconds. Wolf spiders of the genus *Pardosa* and *Alopecosa* were used as prey. *Lampona* and *Drassodes* were placed individually in plastic cups (diameter 3.5 cm, height 5 cm) with gypsum on the bottom and walls covered with a layer of butter to prevent escape. Each prey was introduced after 1 hr of acclimation, and the recording of the hunt was made. In total, 23 hunting sequences involving *Lampona* and 21 sequences involving *Drassodes* were obtained. From these sequences, the following types of behavior were distinguished: **approach**—the prey or the predator moved toward the other; **orientation**—the predator turned to face the direction in which the prey was situated; **pursuit**—the predator pursued the moving prey; **immobile**—the predator stopped on the spot and remained briefly without any other activity; **grasping**—the predator grabbed the prey by means of dense hairs (scopula) on the tarsus and metatarsus; **wrapping**—the predator ran around the prey and released silk, immobilizing the prey in the process; **bite**—the predator delivered a bite to the prey; **holding**—the prey was held by the predator with the first and second pair of legs until it was paralyzed; **feeding**—the predator started consuming the prey. Using this ethogram, transition matrices were created with JWatcher software (Blumstein, Evans, & Daniels, 2006). Then, flow diagrams for each species were created from ethograms and transition probabilities. The frequencies of bites on different body parts (leg or body) were compared using the chi‐square test. To measure the stereotypy of hunting behavior, we used Shannon entropy. Entropy estimates along with 95% confidence intervals were calculated from the transition matrices by the bootstrap method with 1000 simulation runs for both *Lampona* and *Drassodes*.

### 2.5 Morphology of trophic traits

Ten individuals of *Lampona* and *Drassodes* used in laboratory trials were thereafter stored in 75% alcohol and used to measure morphological traits: the length of the prosoma; the length of both segments of the chelicerae; the length of the posterior and anterior spinnerets; the length of the tarsus and metatarsus of the first pair of legs; the area and density of scopula hairs; and the thickness of the cuticle on the prosoma, the opisthosoma, and the legs (femur). Thickness of the cuticle was measured from thin tissue cuts prepared with scalpel. The cuts were made perpendicularly to the surface in the central section of the prosoma, opisthosoma, and femur. LEICA EZ5 and OLYMPUS CX31 stereomicroscopes were used for measurement, both equipped with an ocular micrometer. The obtained data were modeled by means of a GLM model with gamma distribution and a logarithmic link (Pekár & Brabec, 2016).

### 2.6 Prey consumption

The consumption time and net weight gain were observed in eight individuals of *Lampona* and ten individuals of *Drassodes*. Lycosid spiders were used as prey. After capture of the prey, the individuals were checked every 15 min to determine whether they were feeding on the prosoma or opisthosoma of the prey. Feeding on the leg was considered as feeding on the prosoma. All predator and prey individuals were weighed using a Sartorius balance with a precision of 0.001 mg before and after feeding. Prey consumption time was modeled by means of a GLM model with gamma distribution and a logarithmic link (Pekár & Brabec, 2016). The type of predator and consumed body part were used as factors, and the initial weights of both the predator and the prey were used as covariates. The proportion of individuals feeding on the prosoma of the prey throughout the whole time of consumption was modeled using a logit model with binomial distribution and GEE due to the occurrence of paired measurements. The working correlation structure was exchangeable. Net weight gain was analyzed in a similar manner as prey consumption time. The type of predator was used as a factor, and the weights of both the predator and the prey were used as covariates.

## 3 Results

### 3.1 Trophic niche

**Figure 2:**
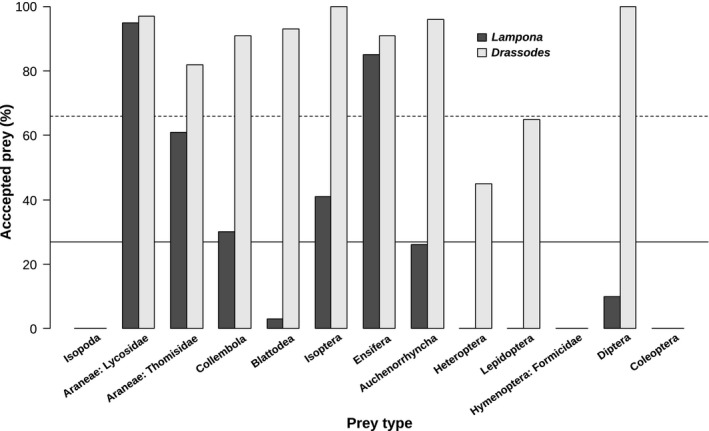
Comparison of the relative frequencies (percentage) of prey accepted by *Drassodes* and *Lampona* in the laboratory. Full horizontal line shows the overall mean of prey acceptance for *Lampona*, dashed line for *Drassodes*

*Lampona* accepted eight of thirteen offered prey types in the laboratory. The average prey acceptance of all thirteen prey types was 27%. It did not accept woodlice, ants, beetles, true bugs, or caterpillars. At a significantly lower frequency, it accepted cockroaches and fruit flies (contrasts, *p* < .01). Termites were accepted at a slightly but significantly higher frequency than average (contrast, *p* = .02). Three prey types were accepted at a significantly higher frequency: crickets, wolf spiders, and crab spiders (contrasts, *p* < .0001) (Figure 2).

*Drassodes* accepted ten of thirteen offered prey types. The average prey acceptance of all thirteen prey types was 66%. It did not accept woodlice, ants, or beetles. Eight prey types were accepted at a significantly higher frequency: springtails, cockroaches, termites, crickets, leafhoppers, fruit flies, wolf spiders, and crab spiders (contrasts, *p* < .001). Only true bugs were accepted at a significantly lower frequency (contrast, *p* = .001).

Levins’ index of niche breadth for *Drassodes* was high (*B*_*A*_ = 0.61), while the niche of *Lampona* was narrow (*B*_*A*_ = 0.23). The niches of both spiders overlapped to a significant extent (*O*_*jk*_ = 0.81).

**Figure 3:**
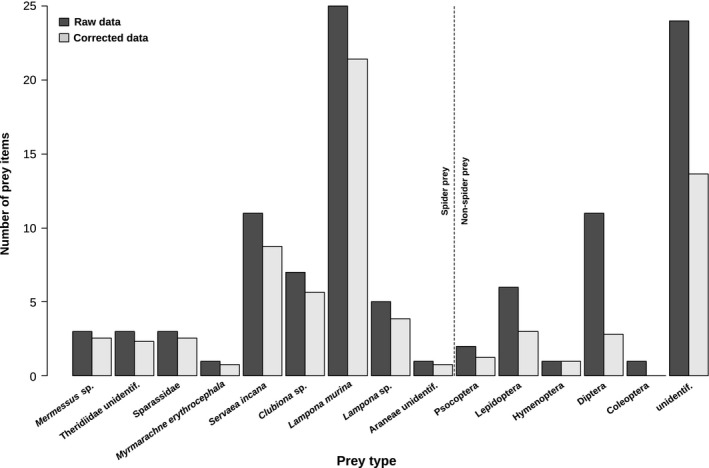
Natural prey in the gut of 36 *Lampona* individuals obtained from DNA analysis

Natural prey DNA fragments were successfully amplified from the guts of 36 of 45 *Lampona* individuals. Spiders formed a major part of the prey (Figure 3, Table S1). The number of spiders was significantly higher compared to other prey pooled (χ^2^_1_ = 9.6, *p* = .002). A high number of the spider prey were conspecifics or congenerics (*Lampona murina* or *Lampona* sp.). The realized niche breadth of *Lampona* was even narrower (*B*_*A*_ = 0.16) compared to the fundamental niche breadth.

### 3.2 Capture efficiency and capture behavior

**Figure 4:**
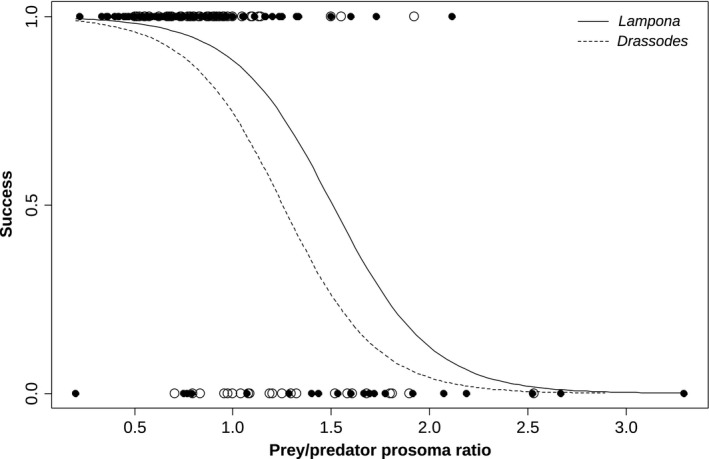
Comparison of the capture success of *Drassodes* and *Lampona* for prey of various relative sizes. Estimated logit models are shown

Capture success with respect to spider prey decreased with the relative prey/predator size ratio both in *Lampona* and *Drassodes* (GEE, χ^2^_1_ = 32.5, *p* < .0001), but it differed between them (GEE, χ^2^_1_ = 4.9, *p* = .03). *Lampona* was more successful in handling larger spider prey than *Drassodes*. *Lampona* achieved 50% capture success with prey approximately 1.5 times larger than itself, while *Drassodes* achieved the same success with smaller prey, 1.25 times larger than itself (Figure 4).

**Figure 5:**
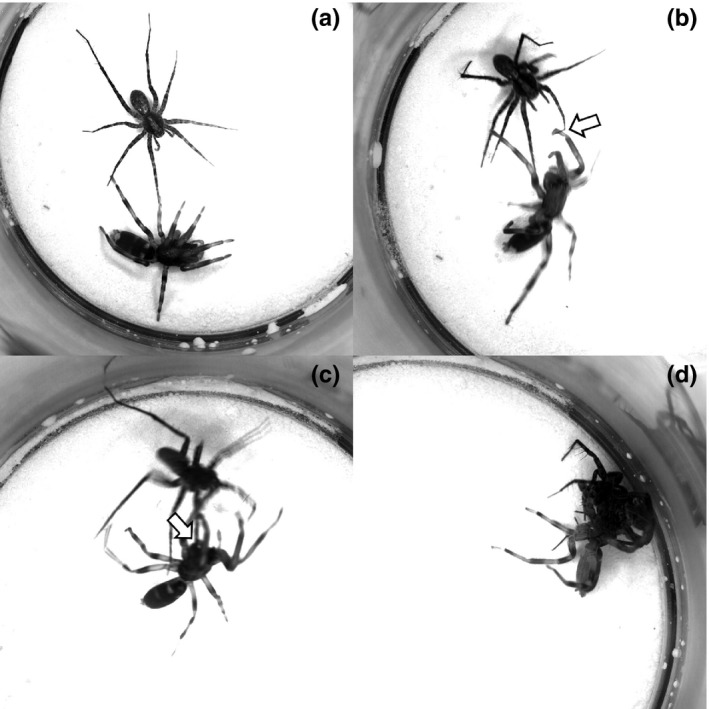
Elements of the predatory behavior of *Lampona* in detail. (a) *Lampona* turns toward the approaching prey and raises the first and second pairs of legs. (b) *Lampona* grabs a leg of the prey with scopulae on the tarsus and metatarsus (arrow). (c) The prey is bitten on the leg (arrow). (d) The prey is held with the first and second pair of legs until it is paralyzed

**Figure 6:**
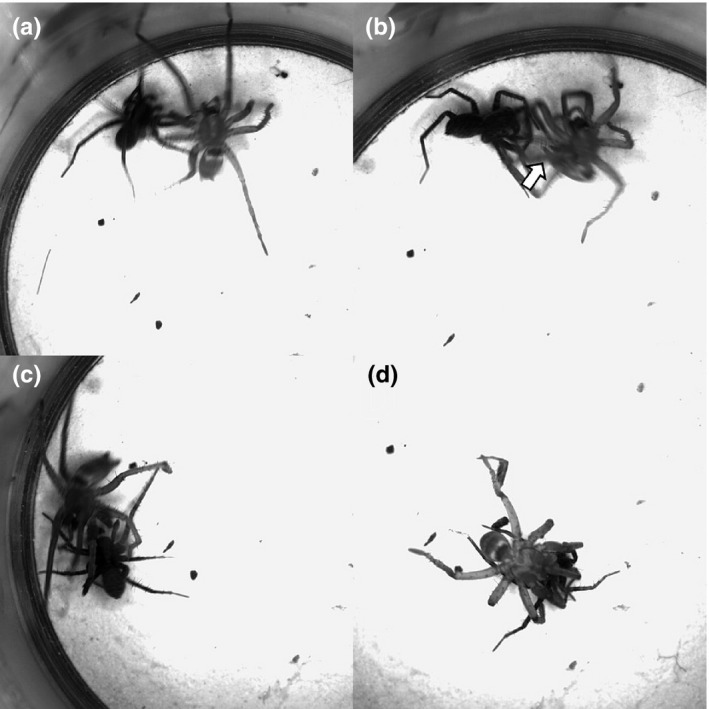
Elements of the predatory behavior of *Drassodes* in detail. (a) *Drassodes* approaches the prey. (b) *Drassodes* turns its abdomen and spinnerets toward the prey (arrow). (c) *Drassodes* runs around the prey and releases silk immobilizing the prey in the process. (d) Afterward, the immobilized prey is killed by a bite

**Figure 7:**
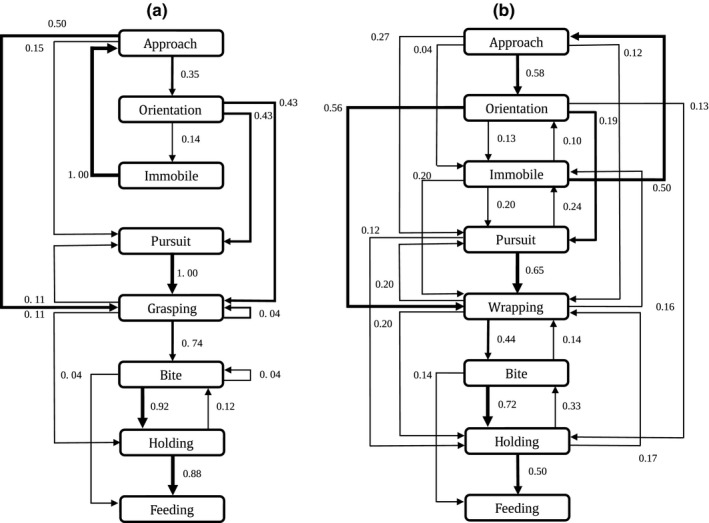
Flow diagrams for *Lampona* (a) and *Drassodes* (b). Transition probabilities are shown for each transition

The key element in the predatory behavior of *Lampona* was grabbing the prey with the scopula on its tarsi and metatarsi. The prey was first grabbed by the predator's forelegs, then bitten, and held firmly until it was paralyzed (Figure 5, Video S1). *Drassodes* used a different tactic: It wrapped the prey in silk to immobilize it (Figure 6, Video S2). The probabilities of transitions during hunting sequence are illustrated in flow diagrams (Figure 7). *Lampona* was more precise in selecting the location of the bite. The prey was bitten more often on the legs (79%, *N* = 24) than on the body (prosoma or opisthosoma) (χ^2^_1_ = 8.7, *p* = .01). *Drassodes* bit the prey more often on the body than on the legs (64%, *N* = 14); however, this difference was not significant (χ^2^_1_ = 1.2, *p* = .29). The Shannon entropy of behavioral sequences differed significantly between *Lampona* and *Drassodes*: The entropy estimate for *Lampona* sequences was 3.93 (95% confidence interval 3.24 ‐ 5.29), while for *Drassodes,* it was 7.08 (95% confidence interval 6.60 ‐ 8.69); therefore, the behavior of *Lampona* was more stereotypical.

### 3.3 Morphology of trophic traits

The cuticle on the prosoma in *Lampona* was 2.5 times thicker than in *Drassodes* (GLM, *F*_1,17_ = 24.5, *p* < .0001, Table 2). The thickness of the cuticle on the opisthosoma and the femur of the first leg did not differ significantly (opisthosoma: GLM, *F*_1,17_ = 0.2, *p* = .66; femur I: GLM, *F*_1,17_ = 2.5, *p* = .13). The anterior spinnerets were longer in *Drassodes* compared to *Lampona* (GLM, *F*_1,17_ = 40.3, *p* < .0001). The length of the posterior spinnerets did not differ significantly between the species (GLM, *F*_1,17_ = 0.0, *p* = .98). The basal segment of the chelicerae was longer in *Drassodes* (GLM, *F*_1,17_ = 46.7, *p* < .0001); also, the length of the fang of the chelicerae was slightly longer in *Drassodes* than in *Lampona* (GLM, *F*_1,17_ = 4.3, *p* = .10). The area of the scopula on the tarsus and metatarsus of the first leg did not differ between *Lampona* and *Drassodes* (tarsus I: GLM, *F*_1,16_ < 0.1, *p* = .94; metatarsus I: GLM, *F*_1,16_ = 1.4, *p* = .26), nor did its density (GLM, *F*_1,16_ = 0.1, *p* = .81, Table 2).

**Table 2:** Comparison of selected morphological traits in *Drassodes* and *Lampona*

Trait	*Drassodes*	*Lampona*
	Mean ± *SD*	Mean ± *SD*
Cuticle thickness (mm)		
Prosoma	0.01 ± 0.005	0.04 ± 0.015
Opisthosoma	0.02 ± 0.004	0.02 ± 0.008
Femur I	0.01 ± 0.004	0.01 ± 0.006
Length of spinnerets (mm)		
Anterior	0.9 ± 0.318	0.53 ± 0.083
Posterior	0.62 ± 0.249	0.67 ± 0.223
Length of chelicerae (mm)		
Fang	0.63 ± 0.237	0.53 ± 0.167
Basal segment	1.32 ± 0.502	1.06 ± 0.235
Scopula area (mm^2^)		
Tarsus I	0.25 ± 0.149	0.26 ± 0.138
Metatarsus I	0.28 ± 0.201	0.34 ± 0.176
Scopula density (no. of hair/0.1 mm)	4.1 ± 0.738	5.3 ± 0.949

### 3.4 Prey consumption

**Figure 8:**
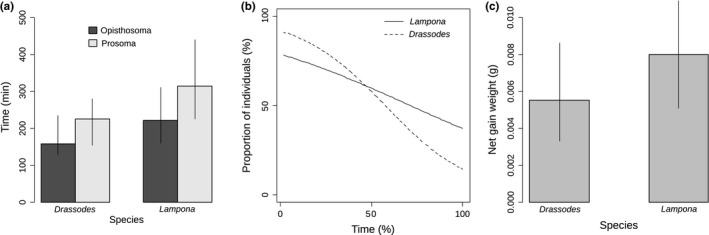
Consumption of the prey. (a) Comparison of the mean consumption time for two body parts of the prey in *Lampona* and *Drassodes*. Vertical lines represent 95% confidence intervals. (b) Change in the proportion of individuals feeding on the prosoma of the prey during the whole consumption period in *Lampona* and *Drassodes*. (c) Comparison of the mean net weight gain for *Lampona* and *Drassodes*. Vertical lines represent 95% confidence interval

Both *Lampona* and *Drassodes* fed longer on the prosoma of the prey than on the opisthosoma (GLM, *F*_1,34_ = 3.9, *p* = .06), but the time spent feeding on each part of the prey differed between the spiders: It was significantly shorter for *Drassodes* (GLM, *F*_1,33_ = 5.8, *p* = .02). Feeding time was significantly influenced by interaction between the initial weights of the predator and of the prey (GLM, *F*_1,30_ = 12.5, *p* = .001) (Figure 8a). The proportion of individuals feeding on the prosoma of the prey changed significantly over the whole consumption period (GEE, χ^2^_1_ = 10.0, *p* = .001); however, it did not differ between *Lampona* and *Drassodes* (GEE, χ^2^_1_ < 0.1, *p* = .64) (Figure 8b). The net weight gain differed between *Lampona* and *Drassodes* (GLM, *F*_1,16_ = 5.9, *p* = .03): The mean net weight gain was 1.5 times higher for *Lampona* than *Drassodes* (Figure 8c). The net weight gain was also influenced by the initial weight of the predator (GLM, *F*_1,15_ = 18.3, *p* = .001) and interaction between the predator and prey weights (GLM, *F*_1,13_ = 7.9, *p* = .02).

## 4 Discussion

As expected, *Lampona* is an araneophagous specialist as it accepted spiders more frequently than other prey, similar to other araneophagous species studied so far (Cerveira & Jackson, 2005; Guseinov, Cerveira, & Jackson, 2004; Jackson & Blest, 1982; Kloock, 2001; Li, Jackson, & Barrion, 1999; Pekár et al., 2011). *Lampona* accepted a few other insect species, although at a lower frequency. By contrast, *Drassodes* did not show a preference for any prey; thus, our results support our expectation it is a euryphagous generalist.

In nature, *Lampona* hunted mainly spiders, as confirmed by the DNA analysis of gut contents. Surprisingly, a large proportion of natural prey was composed of spiders of the same genus or even the same species. Yet cannibalism is not uncommon among araneophagous spiders in the form of sexual or juvenile cannibalism, or oophagy (Clark & Jackson, 1994; Clark, Jackson, & Waas, 1999; Jackson & Pollard, 1997). We confirmed that some of the sequences which were assigned to *Lampona* belonged indeed to the prey and not to the studied predators, because there were three different haplotypes, differing at least by four bases. In addition to the spider prey, we also recorded several other insect prey types. Yet we were not able to identify a considerable proportion of sequences even to order level, as they were assigned to two or more different insect orders with an identical percentage of sequence identity. This may have been because of rapid prey DNA degradation in predators’ guts. Perhaps the prey items had been eaten accidentally much earlier than the nondegraded prey or had been eaten by predators which were subsequently consumed by *Lampona*. Studies on detection of prey DNA in the guts of invertebrate predators showed secondary predation represents a potential significant source of error in the interpretation of data (Hosseini, Schmidt, & Keller, 2008; Sheppard et al., 2005). Therefore, we assume the presence of this DNA is most probably the result of the secondary predation.

Specialized *Lampona* was often able to overcome larger prey than the generalist *Drassodes*, which is in agreement with other studies comparing specialist and generalist predators (Mori & Vincent, 2008; Yamada & Boulding, 1998). Specialists may hunt larger prey in order to decrease the number of risky foraging events when hunting dangerous prey such as spiders. The capture of bigger prey was also observed in other araneophagous spiders (e.g., Eberhard, 1983; Penney & Gabriel, 2009). Here, we provide evidence that a specialist predator is more successful in hunting larger dangerous prey than a generalist.

The ability to hunt larger prey in specialists is facilitated by specialized adaptations. Indeed, *Lampona* possesses a number of morphological adaptations for handling spider prey. It first uses two pairs of legs equipped with scopulae on the tarsus and metatarsus to capture prey. These adhesive patches are an effective hunting tool and represent an alternative strategy to the use of costly silk common in other cursorial spiders (Wolff, Nentwig, & Gorb, 2013). The capture strategy is similar to that used by other araneophagous spiders of the genus *Palpimanus* (Pekár et al., 2011). Furthermore, *Lampona* has a thicker cuticle on the prosoma compared to the euryphagous *Drassodes*. As *Lampona* typically holds the prey under its armored prosoma after the attack, injury resulting from counter attack by the prey is prevented. Similarly, the cuticle of *Palpimanus* is thick and used as armor. Adhesive patches with a similar function to scopulae in *Lampona* can also be found in other araneophagous spiders (Foelix, Jackson, Henksmeyer, & Hallas, 1984), which indicates common traits among araneophagous spiders. The generalist *Drassodes* used a different strategy to hunt prey—immobilization with silk, followed by biting. Morphological comparison revealed the anterior spinnerets of *Drassodes* to be longer than those of *Lampona*. These spinnerets have piriform glands which are used by spiders of the family Gnaphosidae to hunt prey (Deeleman‐Reinhold, 2001). The hunting tactics of *Drassodes* are probably used to hunt dangerous or large prey in general.

Specialists should be more successful than generalists, as they are better adapted to subduing specific prey or foraging in a specific environment and, therefore, they are more precise. This was confirmed in a comparison of specialist and generalist garter snakes foraging in water (Drummond, 1983). Moreover, in the case of dangerous prey, any mistakes could have a significant impact on predator survival (Mukherjee & Heithaus, 2013). As a result, specialization may lead to greater precision in prey capture (Ferry‐Graham et al., 2002). *Drassodes* usually spent more time subduing the prey, as the hunting sequences were longer due to the occasional repetition of behaviors. The strategy of *Lampona*, as a specialist, was more stereotypical and precise. Precision also plays an important role in the strategy of other araneophagous spiders, like those of the genus *Portia*, who choose the location of the strike depending on the dangerousness of the particular prey (Harland & Jackson, 2006).

A specialist should also maximize the utilization of the prey (Townsend et al., 2003). Indeed, *Lampona* spent more time feeding and gained more nutrients in comparison with *Drassodes*. Feeding on one prey type during the life cycle may lead to challenges with respect to limitations on the sources of nutrients. This issue may be resolved by selective feeding on body parts of a prey with different content of proteins and lipids (Pekár, Mayntz, Ribeiro, & Herberstein, 2010). However, *Lampona* did not select only one body part; it fed both on the prosoma and opisthosoma without distinction. Alternatively, nutrient variability may be maintained by the occasional consumption of alternative prey, as araneophagous spiders sporadically hunt insects. Proteins contained mainly in the prosoma seem to be a more important component in the diet of spiders (Blamires, Hochuli, & Thompson, 2009; Mayntz & Toft, 2001). *Lampona* usually began feeding on the prosoma of spider prey and moved to the ophistosoma later. *Drassodes* consumed the prey in a similar way. Other spider generalists consuming insect prey fed in a similar manner (Haynes & Sisojevi, 1966; Kim, Krafft, & Choe, 2005; Pollard, 1989). Alternatively, choice of the prosoma may be connected with mechanics of feeding. The hard prosoma is more suitable for suction than the softer opisthosoma that may collapse under suction pressure (Pollard, 1989, 1990). Spiders may represent a high‐quality food source for other spiders in general (Wise, 1995). Therefore, spiders probably represent an optimal prey for all spiders. A study on the araneophagous *Portia quei* (Toft, Li, & Mayntz, 2010) revealed it to be a behavioral specialist but a metabolical generalist, as it shows versatility in the utilization of prey with varied proportions of nutrients, which is a trait more inherent in euryphagous generalists. Behavioral adaptations for hunting spiders may have evolved without metabolical restrictions and without sacrificing the possibility of utilizing alternative prey.

In conclusion, predators specialized on dangerous prey possess adaptations allowing them to better handle their focal prey. Generalist predators, that is, those which occasionally hunt similar prey, may also possess alternative generalized adaptations for handling dangerous prey. However, the effectiveness of such generalized adaptations will be lower than the effectiveness of more specialized ones.

## Conflict of interest

None declared.

## Data accessibility

Sequences used for designing primers are archived in the GenBank sequence database. Prey sequences obtained from *L. murina* are available in Online Supporting Information (Table S2).
