# Supplementary material for: Capture efficiency and trophic adaptations of a specialist and generalist predator: A comparison
Source: Ecol Evol. 2017 Mar 21;7(8):2756–66. doi: 10.1002/ece3.2812 (PMC5395461; doi:10.1002/ece3.2812)
Supplement: Supplementary file 1 [file ECE3-7-2756-s001.docx]

**Table S1.** List of natural prey detected in the gut of 36 *Lampona* individuals. Summarized from binary data (the presence of a prey sequence type in *Lampona* individuals) transfered from sequence data (Table S2). **N** = total number of prey; **Primary prey** = prey detected without any other predator in one *Lampona* individual; **Secondary prey** = prey detected with other predator(s) in one individual; **Correction coefficient** = relative frequency from acceptance trials; **Secondary prey corrected** = Secondary predation multiplied by the correction coefficient; **N corrected** = sum of Primary prey and Secondary prey corrected.

| **Order** | **Prey** | **N** | **Primary prey** | **Secondary prey** | **Correction coefficient** | **Secondary prey corrected** | **N corrected** |
| --- | --- | --- | --- | --- | --- | --- | --- |
| Araneae | *Mermessus* sp. | 3 | 1 | 2 | 0.775 | 1.55 | 2.55 |
|  | Theridiidae unidentif. | 3 | 0 | 3 |  | 2.325 | 2.325 |
|  | Sparassidae unidentif. | 3 | 1 | 2 |  | 1.55 | 2.55 |
|  | *Myrmarachne erythrocephala* (L. Koch, 1879) | 1 | 0 | 1 |  | 0.775 | 0.775 |
|  | *Servaea incana* (Karsch, 1878) | 11 | 1 | 10 |  | 7.75 | 8.75 |
|  | *Clubiona* sp. | 7 | 1 | 6 |  | 4.65 | 5.65 |
|  | *Lampona murina* L. Koch, 1873 | 25 | 9 | 16 |  | 12.4 | 21.4 |
|  | *Lampona* sp. | 5 | 0 | 5 |  | 3.875 | 3.875 |
|  | Araneae unidentif. | 1 | 0 | 1 |  | 0.775 | 0.775 |
| Psocoptera | Ectopsocidae unidentif. | 1 | 1 | 0 | 0.26 | - | 1 |
|  | unidentif. | 1 | 0 | 1 |  | 0.26 | 0.26 |
| Lepidoptera | *Guestia uniformis* (Meyrick, 1886) | 4 | 2 | 2 | 0 | 0 | 2 |
|  | *Cleora repetita* (Butler, 1882) | 1 | 0 | 1 |  | 0 | 0 |
|  | *Opogona* sp. | 1 | 1 | 0 |  | - | 1 |
| Hymenopetra | Braconidae unidentif. | 1 | 1 | 0 | - | - | 1 |
| Diptera | *Drosophila* sp. | 1 | 0 | 1 | 0.09 | 0.09 | 0.09 |
|  | *Chalarus* sp. | 1 | 0 | 1 |  | 0.09 | 0.09 |
|  | *Dasyhelea* sp. | 1 | 0 | 1 |  | 0.09 | 0.09 |
|  | Ceratopogonidae unidentif. | 1 | 0 | 1 |  | 0.09 | 0.09 |
|  | Cecidomyiidae unidentif. | 1 | 0 | 1 |  | 0.09 | 0.09 |
|  | Chironomidae unidentif. | 1 | 1 | 0 |  | - | 1 |
|  | Diptera unidentif. | 5 | 3 | 2 |  | 0.18 | 2.18 |
| Coleoptera | Curculionidae unidentif. | 1 | 0 | 1 | 0 | 0 | 0 |
| unidentif. | Arthropoda unidentif. | 24 | 10 | 14 | 0.26 | 3.64 | 13.64 |

**Table S2.** Prey sequences obtained from *L. murina*. **No. of reads** = total number of sequences assigned to individual predators; **Valid seq.** = number of sequences without stop codons and reading frame shifts, appearing more than once; **Prey** = prey sequences, other predators found in *Lampona* guts are in bold; **Predator** = sequences assigned to the same haplotype as *Lampona* individual.

| ***Lampona* individual** | **No. of reads** | **Valid**  **seq.** | **Prey** | **Predator** |
| --- | --- | --- | --- | --- |
| Fem 1 (= hapl 2) | 831 | 56 | ***Lampona murina*, hapl 1 (56 seq.)** | - |
| Fem 2 (= hapl 1) | 9,084 | 97 | - | 97 seq. |
| Fem 3 (= hapl 1) | 8,048 | 695 | Insecta * sp. 1 (36 seq.) | 659 seq. |
| Fem 4 (= hapl 1) | 7,334 | 793 | ***Clubiona* sp. (2 seq.)** | 791 seq. |
| Fem 5 (= hapl 2) | 366 | 32 | ***Lampona murina*, hapl 1 (32 seq.)** | - |
| Fem 7 (= hapl 2) | 13,680 | 1,021 | ***Servaea incana* (25 seq.)**  ***Lampona murina*, hapl 1 (17 seq.)**  Insecta * sp. 2 (515 seq.)  Insecta * sp. 1 (409 seq.)  Pipunculidae-*Chalarus* sp. (46 seq.)  *Drosophila* sp. (9 seq.) | - |
| Fem 8 (= hapl 1) | 8,362 | 837 | **Sparassidae (2 seq.)** | 835 seq. |
| Fem 9 (= hapl 1) | 6,125 | 902 | - | 902 seq. |
| Fem 10 (= hapl 2) | 743 | 68 | ***Lampona murina*, hapl 1 (5 seq.)**  ***Servaea incana* (4 seq.)**  Insecta * sp. 1 (2 seq.) | 57 seq. |
| Juv 1 (= hapl 2) | 7,269 | 307 | ***Servaea incana* (25 seq.)**  ***Lampona murina*, hapl 1 (8 seq.)**  Insecta * sp. 1 (224 seq.)  *Guestia uniformis* (8 seq.) | 42 seq. |
| Juv 2 (= hapl 2) | 156 | 26 | ***Lampona murina*, hapl 1 (26 seq.)** | - |
| Juv 4 (= hapl 2) | 43 | 4 | ***Lampona murina*, hapl 3 (4 seq.)** | - |
| Juv 5 (= hapl 1) | 2,200 | 169 | ***Lampona* sp. (41 seq.)**  ***Clubiona* sp. (23 seq.)**  ***Servaea incana* (12 seq.)**  **Linyphiidae - *Mermessus* sp. (6 seq.)**  ***Lampona murina*, hapl 2 (2 seq.)** *Guestia uniformis* (33 seq.) | 52 seq. |
| Juv 6 (= hapl 1) | 3,751 | 496 | - | 496 seq. |
| Juv 7 (= hapl 2) | 15,153 | 935 | ***Lampona murina*, hapl 1 (19 seq.)**  *Guestia uniformis* (916 seq.) | - |
| Juv 8 (= hapl 1) | 4,522 | 493 | Insecta * sp. 1 (8 seq.) | 485 seq. |
| Juv 9 (= hapl 1) | 5,245 | 732 | *Guestia uniformis* (4 seq.)  Insecta * sp. 1 (4 seq.) | 724 seq. |
| Juv 10 (= hapl 2) | 8,906 | 1,134 | ***Lampona murina*, hapl 1 (18 seq.)**  Braconidae (1,114 seq.) | 2 seq. |
| Juv 11 (= hapl 2) | 87 | 0 | - | - |
| Juv 12 (= hapl 2) | 11,595 | 1,865 | ***Lampona murina*, hapl 1 (1,865 seq.)** | - |
| Juv 13 (= hapl 2) | 17,622 | 4,334 | ***Lampona murina*, hapl 1 (1,509 seq.)**  Insecta * sp. 5 (2,825 seq.) |  |
| Juv 14 (= hapl 1) | 16,557 | 1,182 | ***Lampona murina*, hapl 2 (540 seq.)**  ***Lampona* sp. (67 seq.)**  Insecta * sp. 1 (324 seq.)  Insecta * sp. 7 (207 seq.)  Insecta * sp. 5 (3 seq.)  Insecta * sp.6 (8 seq.) | 33 seq. |
| Juv 15 (= hapl 3) | 3,480 | 278 | ***Lampona murina*, hapl 2 (259 seq.)**  ***Servaea incana* (7 seq.)**  Psocoptera s (7 seq.)  Ceratopogonidae sp.1 (3 seq.)  Insecta * sp. 5 (2 seq.) |  |
| Juv 16 (= hapl 2) | 156 | 15 | ***Lampona murina*, hapl 1 (13 seq.)**  ***Lampona* sp. (2 seq.)** | - |
| Juv 17 (= hapl 1) | 18,522 | 2,546 | - | 2,546 seq. |
| Juv 18 (= hapl 3) | 29 | 0 | - | - |
| Juv 19 (= hapl 1) | 624 | 18 | ***Lampona murina*, hapl 2 (5 seq.)**  Diptera* sp.1 (8 seq.)  Diptera* sp.2 (5 seq.) | - |
| Juv 20 (= hapl 1) | 10,230 | 324 | ***Servaea incana* (41 seq.)**  Diptera* sp. 4 (283 seq.) | - |
| Juv 21 (= hapl 2) | 23,824 | 1,702 | ***Lampona murina*, hapl 1 (1,652 seq.)**  **Theridiidae sp.1 (36 seq.)**  **Theridiidae sp. 2 (14 seq.)** |  |
| Juv 22 (= hapl 2) | 5,308 | 501 | ***Lampona murina*, hapl 1 (232 seq.)**  ***Clubiona* sp. (137 seq.)**  ***Lampona murina*, hapl 3 (50 seq .)**  **Theridiidae sp.1 (6 seq.)**  Insecta * sp. 8 (76 seq.) | - |
| Juv 23 (= hapl 2) | 3,995 | 217 | **Sparassidae (38 seq.)**  ***Servaea incana* (34 seq.)**  ***Lampona* sp. (5 seq.)**  Insecta * sp. 3 (76 seq.)  Insecta * sp. 1 (5 seq.)  Insecta * sp. 6 (12 seq.) | 47 seq. |
| Juv 24 (= hapl 1) | 23,852 | 1,369 | Insecta * sp. 2 (2 seq.) | 1,367 seq. |
| Juv 25 (= hapl 1) | 21,345 | 1,186 | ***Lampona murina*, hapl 3 (808 seq.)**  **Clubiona sp. (365 seq.)**  Arthropoda * (7 seq.)  Curculionidae (3 seq.)  Geometridae - *Cleora repetita* (3 seq.) | - |
| Juv 26 (= hapl 2) | 1,064 | 70 | ***Lampona murina*, hapl 1 (28 seq.)**  ***Clubiona* sp. (11 seq.)**  ***Servaea incana* (6 seq.)** | 25 seq. |
| Juv 27 (= hapl 1) | 40 | 0 | - | - |
| Juv 28 (= hapl 1) | 11,104 | 1,033 | **Linyphiidae - *Mermessus* sp. (68 seq.)**  ***Lampona murina*, hapl 2 (9 seq.)**  ***Servaea incana* (2 seq.)**  ***Clubiona* sp. (2 seq.)**  Cecidomyiidae (2 seq.)  Insecta * sp. 1 (31 seq.) | 919 seq. |
| Juv 29 (= hapl 2) | 77,005 | 4,244 | Insecta * sp. 1 (4,244 seq.) | - |
| Juv 30 (= hapl 1) | 6,077 | 819 | Insecta * sp. 1 (2 seq.) | 817 seq. |
| Juv 31 (= hapl 1) | 8,820 | 784 | - | 784 seq. |
| Juv 32 (= hapl 2) | 823 | 50 | Diptera * sp. 3 (14 seq.)  Tineidae - *Opogona* (12 seq.)  Psocoptera - Ectopsocidae (10 seq.)  Chironomidae (2 seq.) | 12 seq. |
| Juv 33 (= hapl 2) | 1,557 | 192 | **Linyphiidae - *Mermessus* (192 seq.)** | - |
| Juv 34 (= hapl 2) | 3,677 | 418 | ***Lampona murina*, hapl 1 (403 seq.)**  ***Servaea incana* (2 seq.)**  Ceratopogonidae- *Dasyhelea* (7 seq.)  Insecta * sp. 4 (6 seq.) | - |
| Juv 35 (= hapl 2) | 186 | 0 | - | - |
| Male 1 (= hapl 2) | 2,069 | 178 | ***Lampona murina*, hapl 1 (48 seq.)**  **Araneae * (20 seq.)**  ***Myrmarachne erythrocephala* (3 seq.)**  ***Lampona* sp. (3 seq.)**  Diptera * sp. 5 (16 seq.) | 88 seq. |
| Male 2 (= hapl 2) | 1,861 | 77 | **Sparassidae (23 sekv.)**  ***Servaea incana* (18 seq.)**  ***Lampona murina*, hapl 1 (8 seq.)**  ***Clubiona* sp. (2 seq.)**  Insecta * sp.1 (24 seq.) | 2 seq. |
| **TOTAL** | **373,327** | **32,199** | **20,417** | **11,782** |

**Insecta ***:

spp. 1 - 4 = Hemiptera (Miridae, Ciccadelidae), Diptera (Sciaridae, Muscidae) < 95% identity

sp. 5 = Coleoptera (Oedemeridae), Diptera (Sciaridae), Tettigoniidae, Mantidae, Anthocoridae < 95% ident.

sp. 6 = Diptera (Mycetophilidae, Sciaridae), Coleoptera (Oedemeridae) = 97.4% ident.

sp. 7 = Diptera (Ephydridae, Tachinidae, Pipunculidae, Empididae, Fanniidae), Psocoptera = 97.5% ident.

sp. 8 = Hemiptera (Psyllidae), Lepidoptera (Nymphalidae) = 87% ident.

**Diptera ***:

sp. 1 = Sciaridae, Drosophilidae = 98.9% ident.

spp. 2,3 = Sciaridae, Agromyzidae, Mycetophiliidae, Anthomyiidae, Scatophagidae, Muscidae = 97.5%

sp. 4 = Mycetophilida, Tachinidae, Ceratopogonidae, Chironomidae = 96.5% ident.

sp. 5 = Muscidae, Sciaridae < 95% ident.

**Araneae *** : Eutichuridae, Tetragnathidae = 93% ident.

**Arthropoda***: Orthoptera, Araneae < 95% ident.
